# First Report of *Vairimorpha* (*Nosema*) *ceranae* in Apiaries of Campeche, Mexico: Molecular Detection and Prevalence

**DOI:** 10.3390/insects16100996

**Published:** 2025-09-25

**Authors:** Arturo Rodríguez-Salinas, Dany Dzib-Cauich, Alberto Santillán-Fernández, José Macias-Macias, Fulgencio Alatorre-Cobos, Álvaro Domínguez Rebolledo, Carlos Castellanos-Zacarías, Carlos Granados Echegoyen, Clemente Lemus-Flores, Alfredo Sánchez-Villarreal, Mauricio Carmona-Arellano, Rosa Us-Camas, Henry Loeza-Concha

**Affiliations:** 1 Maestría en Ciencias Bioprospección y Sustentabilidad Agrícola en el Trópico (BIOSAT), Colegio de Postgraduados, Campus Campeche, Carretera Haltunchén-Edzná, Km 17.5 Sihochac, Champotón C.P. 24450, Campeche, México; artrojib@gmail.com (A.R.-S.);; 2 Tecnológico Nacional de México, Instituto Tecnológico Superior de Calkiní, Calkiní, Av. Ah Canul S/N por Carretera Federal, Campeche C.P. 24900, Campeche, México; 3 SECIHTI-Colegio de Postgraduados, Campus Campeche, Carretera Haltunchén-Edzná, Km 17.5 Sihochac, Champotón C.P. 24450, Campeche, México; 4 Departamento de Ciencias de la Naturaleza, CUSUR, Universidad de Guadalajara, Av. Enrique Arreola Silva No. 883, Colonia Centro, Ciudad Guzmán C.P. 49000, Jalisco, México; joseoc@cusur.udg.mx; 5 SECIHTI-Unidad de Biología Integrativa Centro de Investigación Científica de Yucatán, A.C., Calle 43 No. 130 × 32 y 34, Col. Chuburná de Hidalgo, Mérida 97205, Yucatán, México; 6 Instituto Nacional de Investigaciones Forestales, Agrícolas y Pecuarias, Campo Experimental Mocochá, Km 25 Antigua Carretera Mérida-Motul, Mocochá C.P. 97454, Yucatán, México; alvaroedr@gmail.com; 7 Instituto Tecnológico de Conkal, Av. Tecnológico S/N, Conkal C.P. 97345, Yucatán, México; jacintocastellanos@gmail.com; 8 SECIHTI-Instituto Politécnico Nacional (IPN), Centro Interdisciplinario de Investigación para el Desarrollo Integral Regional (CIIDIR), Hornos No. 1003, Col. Noche Buena, Santa Cruz Xoxocotlán C.P. 71230, Oaxaca, México; cgranadose@ipn.mx; 9 Unidad Académica de Medicina Veterinaria y Zootecnia, Universidad Autónoma de Nayarit, Km 3.5. Carretera Compostela-Chapalilla, Tepic C.P. 63700, Nayarit, México; clemus@uan.edu.mx

**Keywords:** nosemosis, *Nosema ceranae*, PCR, Polyacrylamide Gel Electrophoresis, beekeeping, Mexico

## Abstract

Nosemosis, caused by microsporidians of the genus *Vairimorpha*, is one of the most significant diseases affecting the honey bee *Apis mellifera* L. (Apidae: Hymenoptera), with negative impacts on its health and productivity. This study aimed to assess the presence of *Vairimorpha ceranae* in apiaries from the state of Campeche, Mexico. Honey bee samples were collected from 79 colonies across 29 apiaries, and DNA was extracted for analysis. Detection of the microsporidian parasite was achieved through amplification of the 16S small subunit ribosomal RNA gene by PCR and PAGE, revealing a high prevalence of *Vairimorpha ceranae*. The results represent the first confirmed record of *Vairimorpha ceranae* in this region, with widespread infection detected among sampled colonies. This finding is crucial for identifying the frequency and geographic distribution of this parasite in Mexico and will support the development of targeted management and control strategies adapted to local conditions, ultimately contributing to the conservation and health of honey bee populations in Campeche.

## Simple Summary

This study reports the first confirmed detection of the microsporidian *Vairimorpha ceranae* in *Apis mellifera* colonies from Campeche, Mexico—one of the country’s key honey-producing regions. Samples from various localities were analyzed using Cantwell’s microscopic method, Polymerase Chain Reaction (PCR), and Polyacrylamide Gel Electrophoresis (PAGE). The results revealed a widespread distribution of *Vairimorpha ceranae* across the state. This finding provides essential epidemiological data for understanding nosemosis in southeastern Mexico and underscores the need for targeted sanitary strategies to safeguard honey bee health, a critical component of pollination services and sustainable regional agriculture.

## 1. Introduction

Nosemosis is a significant global disease affecting honey bees (*A. mellifera*), primarily caused by microsporidian pathogens that have been recently reclassified from the genus *Nosema* to *Vairimorpha*, specifically, *V. apis* and *ceranae*, based on molecular phylogenetics [1]. These pathogens are characterized as opportunistic, intracellular, spore-forming parasites [2] and are known to cause significant damage to bee colonies. They are considered major contributors to the global decline in bee populations [3] and have been reported in nearly all countries where beekeeping is practiced [4].

For many years, it was believed that nosemosis in European honey bees was exclusively caused by *V. apis* [2,5,6]. However, recent studies have shown that *V. ceranae*, a species initially infecting the Asian honey bee *A. cerana* F. (Apidae: Hymenoptera) [7], is also capable of parasitizing *A. mellifera* across multiple continents [8,9]. Nosemosis affects the epithelial cells of the midgut (ventriculus), causing digestive disorders that impair nutrient absorption and reduce bee longevity [10]. *V. ceranae*, in particular, has been shown to suppress the immune response in bees, making them more susceptible to secondary infections while also shortening their lifespan, weakening colonies, and increasing winter mortality, all without exhibiting specific clinical signs [3]. Additionally, this species has been associated with colony losses [2,11,12] due to its high pathogenic potential and its greater prevalence in *A. mellifera* compared to *Vairimorpha apis* [2,11]. Notably, *V. ceranae* has been implicated as one of the possible reasons for Colony Collapse Disorder (CCD) [13].

In Mexico, this parasitic infection has been present since the 1960s [8], and for many years, *V. apis* was assumed to be its sole causative agent. However, subsequent investigations identified *V. ceranae* spores in samples collected between 1995 and 1996 from apiaries in central Mexico, confirming its presence in Africanized bee colonies for over 25 years [14].

Traditionally, diagnosis of this disease has relied on Cantwell’s microscopic technique [15], which involves the visual identification of microsporidian spores under light microscopy. However, now that both *V. apis* and *V. ceranae* are known to infect *A. mellifera*, it is crucial to identify the specific species involved in a given region. For this, molecular techniques are essential, as the spores are morphologically indistinguishable under light microscopy [16,17]. Moreover, microscopy has been shown to have limited efficacy in detecting infections with low spore loads, whereas molecular diagnostic methods, such as PCR, offer a more reliable and precise approach for pathogen detection [9].

In recent years, increasing global reports of *Vairimorpha* infections have emerged, with beekeepers reporting colony mortality and reduced productivity. This has led to the hypothesis that the rapid spread of *V. ceranae* may be playing a significant role in this global apicultural crisis [18]. In Mexico, the state of Campeche is one of the leading honey-producing regions, with the municipality of Champotón ranked as the top producer in the state [19]. Beekeeping is a vital source of income for many rural communities in the region and is often practiced in conjunction with other economic activities. Despite its economic and ecological importance, current information regarding the distribution of *Vairimorpha* in Campeche remains scarce. Although recent reports have confirmed its presence in apiaries in Hopelchén and Sihochac [20], no data are available on its presence in other localities, nor has the specific species been confirmed.

In light of this information gap, the objective of this study was to identify, for the first time, the presence of *V. ceranae* in apiaries across the state of Campeche using molecular methods. The results provide evidence of its distribution in this region of southeastern Mexico and contribute essential data for epidemiological surveillance and the future development of sanitary management strategies in the regional beekeeping sector.

## 2. Materials and Methods

### 2.1. Study Area

**Figure 1:**
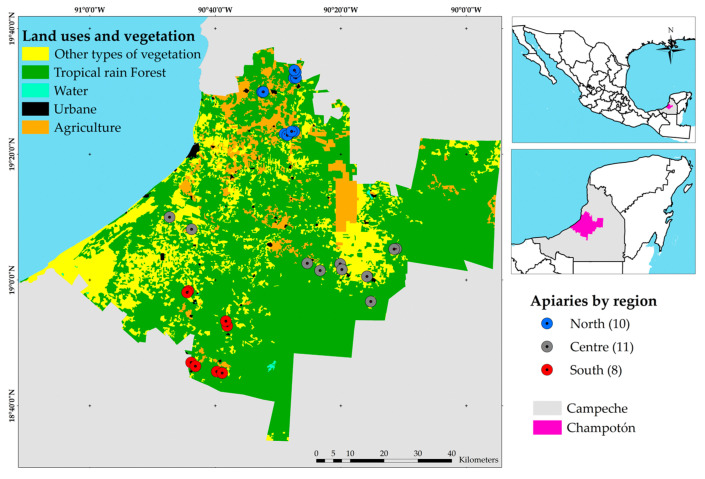
Land use, vegetation cover, and distribution of apiaries in Champotón, Campeche, Mexico.

This study was conducted using honey bee (*A. mellifera*) samples collected from apiaries located in various localities of the municipality of Champotón, Campeche, Mexico (Figure 1). Sampling was carried out between September 2023 and January 2024, immediately following the rainy season, a period during which increased infection levels caused by *Vairimorpha* spp. have previously been documented [5].

The samples were obtained from three geographical zones: north, central, and south. Colonies from a total of 29 apiaries, distributed across these three zones of Champotón, were analyzed to evaluate and identify the prevalence of *V. ceranae*. In the northern zone, ten apiaries located in the communities of Sihochac, Hool, and Arellano were included. The central zone encompassed eleven apiaries across the localities of Vicente Guerrero, Chaccheito, Miguel Allende, Chilam Balam, Módulo Nuevo Paraíso, and La Providencia. Finally, the southern zone included eight apiaries located in San Pablo Pixtún, General Ortíz Ávila, Miguel Colorado, and Cinco de Febrero.

Stratification of the study area allowed for a more precise analysis of the distribution of *Vairimorpha* spp. infections and potential geographic variations in colony infection levels. In each defined zone (north, central, and south), field visits were conducted to identify active apiaries and contact beekeepers willing to participate. A total of 414 colonies from 29 apiaries were recorded, with an average of 14 colonies per production unit. To ensure statistical representativeness and optimize the analysis, 20% of the colonies from each zone were randomly selected, resulting in a final sample of 79 colonies.

### 2.2. Colony Sampling

For molecular analysis, returning foragers carrying pollen loads and guard bees positioned at the hive entrance, as well as young nurse bees from lateral combs adjacent to brood frames within the brood chambers (10 individuals per colony), were collected and placed into 2 mL plastic microtubes containing DNA/RNA Shield (Zymo Research), then stored at 4 °C until processing. For microscopic analysis, approximately 200 bees were collected in 96% ethanol from the lateral frames of the brood chamber, supers, and hive entrances. These samples were stored at room temperature until analysis.

### 2.3. Microscopic Diagnosis of Nosemosis

Light microscopy was used to detect the presence of *Vairimorpha* spores in bee samples, following the protocol described by Cantwell [15]. Due to the morphological similarity of spores among microsporidian species infecting *A. mellifera*, it was not possible to distinguish species under the microscope; therefore, a complementary molecular analysis was conducted to achieve species-level identification.

### 2.4. DNA Extraction from Vairimorpha Spores

Given that spores could not be reliably distinguished microscopically, DNA was extracted from the abdomens of ten bees per colony, selected as potentially infected for subsequent molecular analysis following the protocol proposed by Hunt [21] and Vázquez [22], with minor modifications. Bees were removed from microtubes containing DNA/RNA Shield (Zymo Research) and dissected to isolate the abdomens, which were ground in liquid nitrogen using a mortar and pestle. The resulting powder was transferred to 2 mL microtubes and stored at −80 °C.

Between 60 and 100 mg of abdominal tissue was placed into 1.5 mL microtube containing 350 μL of lysis buffer (CTAB 0.03 M, Tris 0.05 M (pH 8), EDTA 0.01 M (pH 8.0), and NaCl 0.75 M in sterile ultrapure water). Then, 5 μL of proteinase K (Thermo Scientific^™^, Waltham, MA, USA, 20 mg/mL) and 2 μL of RNase (Thermo Scientific™, Waltham, MA, USA, 10 mg/mL) were added. Samples were incubated at 60 °C for 2 h with inversion every 20 min.

Following incubation, 170 μL of NaCl-Tris solution (1.5 M NaCl, 0.5 M Tris pH 8.0) and 600 μL of phenol:chloroform:isoamyl alcohol (25:24:1, Invitrogen™, Waltham, MA, USA) were added. Tubes were vortexed and incubated at room temperature (RT) for 5 min, then centrifuged at 12,000 rpm for 10 min. The supernatant was transferred to a fresh tube, mixed with an equal volume of chloroform, and centrifuged under the same conditions. DNA was precipitated by adding two volumes of cold 100% ethanol and 1/10 volume of 3 M sodium acetate (pH 5.2), followed by a 10 min incubation at RT. The pellet was washed twice with 75% ethanol and centrifuged at 12,000 rpm for 5 min per wash. Residual ethanol was removed by pipetting, and the DNA pellet was air-dried at RT for 10 min before resuspension in sterile ultrapure water and stored at −20 °C.

DNA quantification was determined using a NanoDrop spectrophotometer (Thermo Scientific™, Waltham, MA, USA), and integrity was assessed by 0.8% agarose gel electrophoresis in 1XTAE buffer, stained with 1 μL of ethidium bromide (10 mg/mL) and visualized under UV light using a Bio-Rad XR+ Gel Documentation System (Hercules, CA, USA).

### 2.5. Detection of Vairimorpha ceranae by PCR and Polyacrylamide Gel Electrophoresis

Amplification of the 16S rRNA of *Vairimorpha* species was carried out using the primers developed by Higes et al. [10], designed to generate 240 bp and 252 bp amplicons specific to *V. apis* and *V. ceranae*, respectively. Primer specificity was validated using BLAST + 2.15.0 (http://www.ncbi.nlm.nih.gov/BLAST/)(accessed on 7 July 2025). The forward primer was NOS-FOR (5′-TGCCGACGATGTGATATGAG-3′), and the reverse primer was modified by adding a guanine to its 3′ end, resulting in NOS-REV (5′-CACAGCATCCATTGAAAACGG-3′).

The PCR reactions were performed in a Bio-Rad T-100 thermocycler™ (Waltham, MA, USA) in a final volume of 30 μL. Each reaction contained 15 μL of DreamTaq Green PCR Master Mix 2 X (Thermo Scientific™, Waltham, MA, USA), 1 μL each of forward and reverse primers (10 μM), a variable volume of ultrapure sterile water, and 150 ng of genomic DNA extracted from approximately 1849 spores. PCR conditions were as follows: initial denaturation at 95 °C for 3 min; 35 cycles of 95 °C for 30 s, 54 °C for 30 s, and 72 °C for 1 min; and a final extension at 72 °C for 5 min.

DNA electrophoresis was carried out in 4% agarose gels with 12 μL of the PCR reaction per well, and a 100 bp molecular weight marker (Invitrogen™, Waltham, MA, USA) was used to estimate the amplicon size. DNA PAGE gel electrophoresis was prepared with polyacrylamide (5%) gels and electrophoresed in 0.5 X TBE buffer at 60 W for 1 h and 50 min. A 10 bp molecular weight marker (Invitrogen™, Waltham, MA, USA) was used to estimate the amplicon size. The gels were stained with silver nitrate and developed using sodium carbonate to enhance contrast for visual interpretation of the amplified DNA fragments.

### 2.6. Statistical Analysis

Statistical analysis was performed to determine whether significant differences existed in the prevalence of *V. ceranae* among the three geographic strata evaluated. Normality assumptions were tested using the Shapiro–Wilk test. Upon detecting non-normal distribution, the non-parametric Kruskal–Wallis test was applied to compare medians across groups. All statistical analyses were conducted using R version 4.2.0 (R Foundation for Statistical Computing, Vienna, Austria).

Additionally, Cohen’s Kappa coefficient was calculated to estimate the degree of agreement between microscopic and molecular diagnostic results, quantifying the diagnostic consistency of both methods. Interpretation of the Kappa values was based on the following thresholds: poor agreement = 0.00–0.20, fair = 0.21–0.40, moderate = 0.41–0.60, good = 0.61–0.80, and very good = 0.81–1.00.

Finally, the diagnostic performance of Cantwell’s microscopic technique was assessed by calculating its sensitivity, specificity, positive predictive value (PPV), and negative predictive value (NPV), using the molecular diagnosis as the reference standard.

## 3. Results

### 3.1. Microscopic Identification of Vairimorpha *spp.*

**Figure 2:**
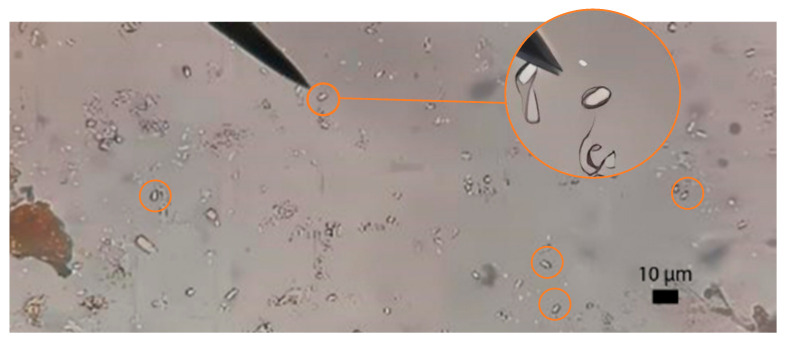
Microscopic diagnosis of *Vairimorpha* spp. using the Cantwell technique (1970). Spores from abdomen macerates were observed in a Neubauer chamber BLAUBRAND^®^ (Wetzlar, Germany). Microscope: Leica CME^®^ (Wetzlar, Germany); Objective: 40×, NA 0.65; Illumination: brightfield. Image captured with a Xiaomi 2201117SY (Beijing, China), smartphone directly on the eyepiece; no digital zoom was used; only uniform linear adjustments (levels/contrast) were applied. Scale = 10 µm. Images are representative of *n* = 79 samples analyzed. Camera on a Redmi Note 11s cell phone, Xiaomi (Beijing, China). In the circle is the microsporidium detected by microscopy.

Microscopic analysis revealed the presence of *Vairimorpha* spp. in the three regions evaluated in the municipality of Champotón, Campeche. Of the 70 samples tested, 39 tested positive, resulting in an overall prevalence of 49.36%. In the northern region, a prevalence of 80% was found, so, in 20 of 25 colonies, the presence of *Vairimorpha* spp. was found; in the central region, the prevalence was 24.24%, with microsporidium found in 8 of 33 colonies evaluated; and in the southern region, the prevalence was 52.38%, so that of 21 colonies evaluated, 11 were positive for *Vairimorpha* spp. (Figure 2).

### 3.2. PCR Amplicon Identification in Agarose Gel

**Figure 3:**
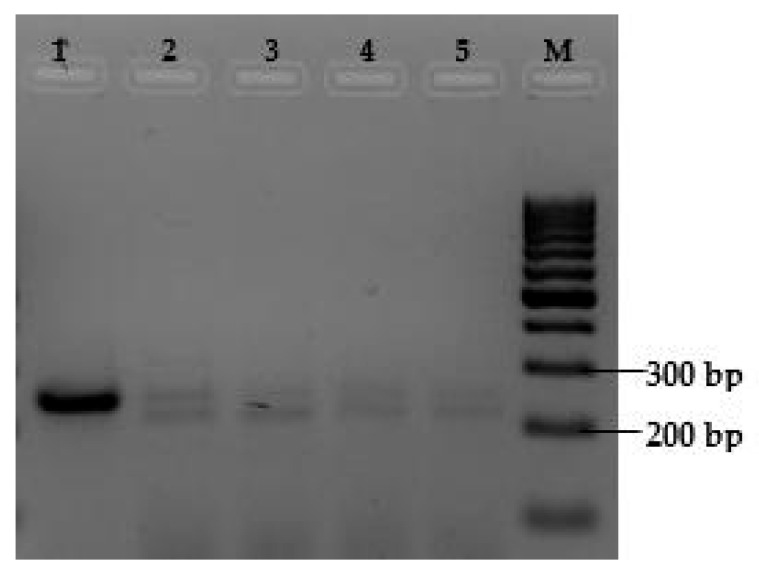
Representative agarose gel electrophoresis (4%) of the PCR from apiary samples. Note that Lane 1 shows a single band corresponding to the expected 252 bp of *V. ceranae,* whereas Lanes 2–5 display two PCR products, one with the exact size of Lane 1 and a smaller amplicon. M denotes 100 bp ladder.

**Figure 4:**
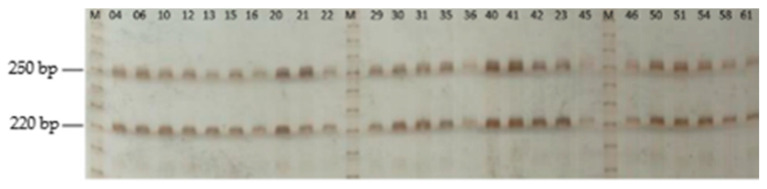
PAGE (5%) of 26 samples. All samples showed PCR amplification of 252 bp and 222 bp amplicons, indicating a positive reaction to *V. ceranae* as well as an unknown target, likely another *Vairimorpha* species yet to be identified. Numbered lanes correspond to the samples, and the letter M indicates the 10 bp molecular marker.

**Figure 5:**
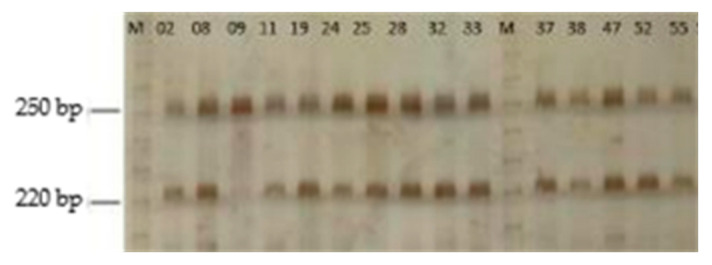
PAGE (5%) of 16 samples. In most of the samples shown, the PCR yielded 252 bp and 222 bp amplicons, indicating a positive reaction to *V. ceranae* as well as an unknown *Vairimorpha* species. Note that a few samples, such as 09, are negative for the unknown 222 bp amplicon but are positive for *V. ceranae*. Numbered lanes correspond to the samples, and the letter M indicates the 10 bp molecular marker.

DNA gel electrophoresis analysis revealed two distinct amplicons generated by the primers targeting the hypervariable region of the 16S rRNA gene of *Vairimorpha* sp. in most of the tested samples (Figure 3). The low resolving power of the 4% agarose gel made it challenging to estimate the size of the observed DNA fragments, particularly the smaller fragment (Figure 3). Therefore, PAGE was used, which has a high resolving power, facilitating the separation of DNA fragments that present length differences of up to a single base pair [23] compared to agarose gels. PAGE allowed us to determine that one of the amplicons had a size of 252 bp, which is consistent with the expected product size of 252 bp for *V. ceranae.* However, the smaller fragment resulted in an unexpected 222 bp amplicon that does not correspond with the reported size of 240 bp of *Vairimorpha* spp. (Figure 4 and Figure 5).

### 3.3. Molecular Identification of Vairimorpha *spp.* and Vairimorpha ceranae

To better understand the distribution of co-infections, the PCR results were analyzed by region. In the northern region, 17 colonies (68%) exhibited co-infection with *V. ceranae* and unidentified Vairimorpha, while 8 colonies (32%) had single infections of *Vairimorpha ceranae*. In the central region, 29 colonies (87.88%) showed co-infection, while 4 (12.2%) were only infected with *V. ceranae*. In the southern region, 17 colonies (80.95%) were co-infected, while 4 (19.05%) showed only *V. ceranae*.

Overall, 100% of the 79 samples tested by molecular methods were found to be positive for *V. ceranae*. Among them, 63 (79.75%) displayed co-infection with *Vairimorpha*, while 16 (20.25%) exhibited single infections of *V. ceranae*.

### 3.4. Agreement Between Diagnostic Techniques

By microscopic analysis, a positive agreement percentage (PPA) = 49.37% (95% CI 40/79) and a negative agreement percentage (NPA) = 50.63% (95% CI 39/79) were observed; in this sense, the sensitivity of the Cantwell technique was 49.37%, and its specificity was 0.0%. The PCR (not sequenced) presented a PPA = 100% (95% CI 79/79); therefore, its sensitivity was 100%, and by not finding discordant data, its specificity was 0.0% (Table 1). In this sense, regarding the Cohen’s Kappa coefficient (95% CI), between the Cantwell microscopic method and the PCR (not sequenced), its concordance was 0%. The microscopic method has proven to be an efficient technique for finding true positives; however, when infestations are mild, they can be associated with false negatives, so its specificity tends to be null. By contrast, PCR (not sequencing) has a positive predictive value of 100% and a negative predictive value of 0%. According to the above, these findings reveal a high rate of false negatives and a limited capacity of microscopy to rule out non-infected cases, which underscores the importance of combining microscopic techniques with molecular tools for the diagnosis of this disease.

## 4. Discussion

Diseases caused by *Vairimorpha* represent one of the most significant threats to global beekeeping, particularly *V. ceranae*, whose rapid spread has been linked to colony losses. In Campeche, Mexico, despite the importance of beekeeping, information on nosemosis is limited and has been based solely on microscopy. This study represents the first molecular report of V. ceranae in the region, providing crucial evidence for its diagnosis and control.

Analysis revealed that the 252 bp fragment is consistent with previous reports by Higes et al. [10] and Tosun and Yaman [23], who reported an expected product size of 252 bp for *V. ceranae*. However, a 222 bp amplicon was also observed, which was unexpected, as *V. apis* is reported by these authors to produce a 240 bp fragment.

Firstly, it is important to note that the 252 bp fragment is shorter than previously reported [10,23]. However, with the primers used, we retrieved 16S *Vairimorpha* sp. sequences from GeneBank at NCBI with identifiers LC510252.1 and MH349843.1, both of which are 252 bp in size, as reported with the primers used. Thus, the presence of *V. ceranae* in apiaries from the state of Campeche is confirmed.

The unexpected 222 bp band suggests a co-infection with either another yet-unknown *Vairimorpha* species or with another unknown organism that probably would be a microsporidian. BLAST analysis showed that the same primer set can amplify fragments of 240 bp from *N. bombi* (AY741104.1), *N. neumanni* (MF882996.1), and *N. apis* (X73894.1) and 244 bp from *Vairimorpha necatrix* (XR_010573990.1) (see Supplemental Figure S1). Therefore, our results suggest either the presence of an unknown and not previously reported species of *Vairimorpha* or a deletion of the 16S gene in any of the previously reported species infecting bees. However, we cannot rule out that the 222 bp corresponds to a yet-unknown organism not represented in the GeneBank nr database. Determining the identity of the 222 bp fragment would require sequencing the fragment and, ideally, the full-length 16S gene. Nonetheless, the results indicate the presence of another organism in a probable co-infection process.

In this context, the results obtained in this study confirm, for the first time, the presence of *V. ceranae* in the state of Campeche, Mexico. Its detection is not unexpected, considering previous reports from multiple regions across the country, including Hidalgo, Morelos, Mexico City [8], Estado de México [14], Jalisco [24], Baja California Norte, and Sonora [3]. This dispersion pattern suggests that *V. ceranae* is widely distributed in Mexico and that its presence in the Yucatán Peninsula indicates successful adaptation to diverse climatic conditions, supporting the hypothesis of an established presence in the national territory [14].

Additionally, the detection of co-infection suggests either the coexistence and adaptation of two closely related pathogens or an evolutionary selection process in which *V. ceranae* has lost a fragment of the 16S rRNA gene without an apparent reduction in infection capacity.

The high prevalence observed in Champotón (100%, including single and mixed infections) aligns with the findings of Guzmán-Novoa et al. [8], who reported a 94% prevalence of *V. ceranae* in central Mexico. Similarly, Ramos-Cuellar et al. [24] documented only the presence of *V. ceranae* in apiaries from Jalisco. However, some regions exhibit contrasting patterns. For instance, in Baja California Norte and Sonora, Cueto et al. [3] reported a prevalence of 87.32% for *V. apis* and only 12.68% for *V. ceranae*. This regional contrast suggests that the distribution of *V. ceranae* in Mexico may be influenced by climatic factors, being more common in warm and humid regions such as the Yucatán Peninsula, whereas *V. apis* may still predominate in arid or semi-dry zones.

International studies support this trend, indicating that *V. ceranae* has gradually displaced *V. apis* as the main causative agent of nosemosis, particularly in areas with mild winters where the temperature rarely falls below 4 °C [25,26,27]. Its ability to establish and persist under warm environmental conditions has been key to its global spread. Notably, a strong correlation has been observed between ambient humidity and infection levels, with relative humidity being one of the most influential factors in its proliferation [23].

Moreover, *V. ceranae* maintains active infections throughout the year, independent of seasonal or climatic events, and its predominance over *V. apis* is attributed to its greater adaptability and lower susceptibility to environmental fluctuations, which provide an evolutionary advantage across diverse regions [28,29]. Its predominance over *V. apis* has been attributed to a greater adaptability to diverse environments and lower susceptibility to seasonal changes, which confer an evolutionary advantage in various parts of the world [28,29]. In this context, *V. ceranae* has been found in regions with high relative humidity and consistently elevated temperatures, such as the Yucatán Peninsula, but also in countries like Saudi Arabia [27], where most of the territory is desertic with hot, dry climates and extreme temperatures, or Italy [30], which has a humid subtropical climate characterized by cool winters and hot summers.

It is important to note that the low concordance between microscopic and molecular diagnoses may be attributed to the limited sensitivity of microscopy for detecting low parasite loads, in contrast to molecular techniques, which offer higher detection sensitivity. This observation is consistent with reports by Guerrero-Molina et al. [14], who identified *V.* ceranae via PCR in samples where spore presence was not observed under microscopy, likely due to quantities falling below the visual detection threshold.

## 5. Conclusions

This study represents the first report of *V. ceranae* in honey bee (*A. mellifera*) colonies from the state of Campeche, Mexico, confirming its presence through molecular diagnosis. The species was detected in 100% of the analyzed samples, either as a single infection or in co-infection, demonstrating a high prevalence in the Champotón region. The observed geographic distribution and infection levels are consistent with reports from other parts of the country, particularly in regions characterized by warm and humid climates.

Its ability to persist under such conditions, along with its detection in colonies lacking visible clinical signs, underscores the importance of strengthening epidemiological surveillance systems using highly sensitive molecular tools. These would enable timely detection and facilitate more effective sanitary management of bee colonies.

## Figures and Tables

**Table 1:** Comparison of microscopy and PCR results (presumptive, unsequenced reference) in 79 samples.

Microscopy	PCR (+)	PCR (−)	Total
**(+)**	39	0	39
**(−** **)**	40	0	40
**Total**			79

This table shows the number of positive (+) and negative (−) samples detected by each method.

## Data Availability

The data is available upon request from the corresponding authors at loeza.jesus@colpos.mx and dadzib@itescam.edu.mx.

## Supplementary Materials

The following supporting information can be downloaded at https://www.mdpi.com/article/10.3390/insects16100996/s1. Figure S1: Alignment of the retrieved sequences of *Vairimorpha*/Nosema from the NCBI database. The alignment shows the region amplified by PCR with the primers used in this study (Higes, Martín & Meana [10]), and in brackets is depicted the position of the primers within the sequences. It can be noted that *N. cerenae* size is bigger, as it introduces a gap between the rest of the sequences. On the other hand, the other four sequences are mostly identical, producing a 240 bp fragment; however, none of the sequences has a 222 bp size like the one shown in Figure 2 and Figure 3. Alignment was performed using the Multiple Sequence Alignment Kalign tool from the EMBL’s European Bioinformatics Institute website (https://www.ebi.ac.uk/) (accessed on 8 July 2025), with standard parameters. GeneBank sequences shown are *N. cerenae* LC510252.1, *N. bombi* AY741104.1, *Vairimorpha neumanni* MF882996.1, *Vairimorpha* LC467309.1, and *Vairimorpha necatrix* XR_010573990.1. Finally, see Figure S2 representative agarose gel electrophoresis (4%) of the PCR from apiary samples can be observed. Note that lane 8 shows a single band corresponding to the expected 252 bp of V. ceranae, lane C (-) is the negative control of PCR reaction (no DNA), whereas lanes 1–7 display two PCR products, one of the exact sizes as lane 8 and a smaller amplicon. M denotes 100 bp ladder.
