# Supplementary material for: First Report of Vairimorpha (Nosema) ceranae in Apiaries of Campeche, Mexico: Molecular Detection and Prevalence
Source: Insects. 2025 Sep 25;16(10):996. doi: 10.3390/insects16100996 (PMC12565220; doi:10.3390/insects16100996)
Supplement: Supplementary file 1 [file insects-16-00996-s001.zip › insects-3817709-supplementary.pdf]

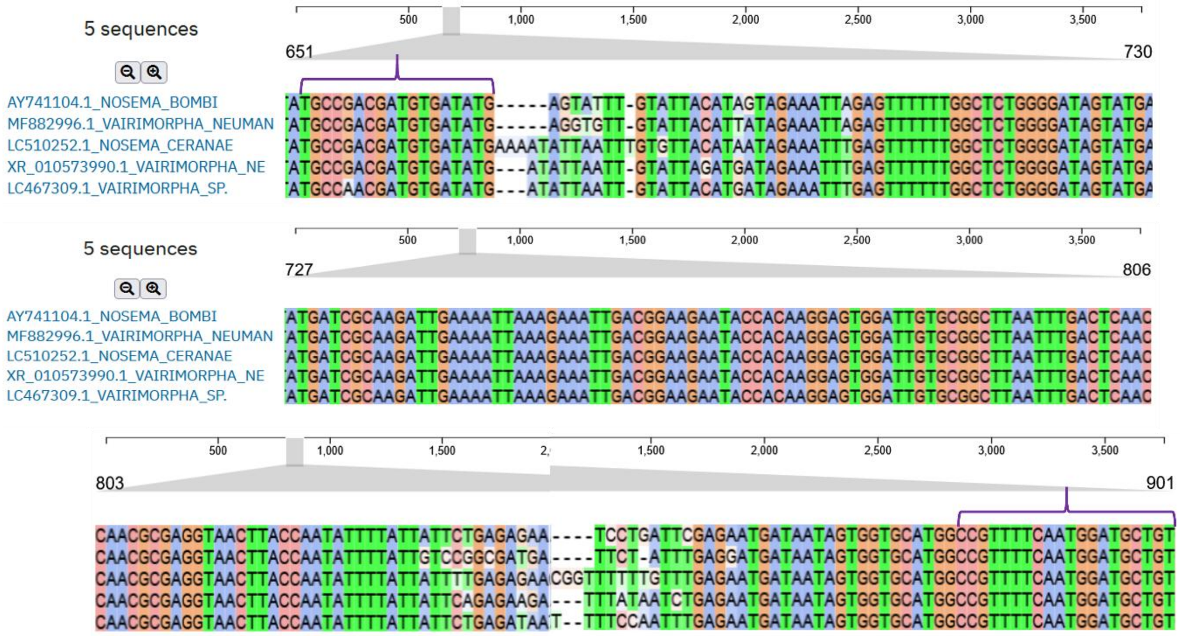

Figure S1. Aligment of the retrieved sequences of VairimorphaNosema from NCBI database. The aligment show the region amplified by PCR with the primers used in this study.

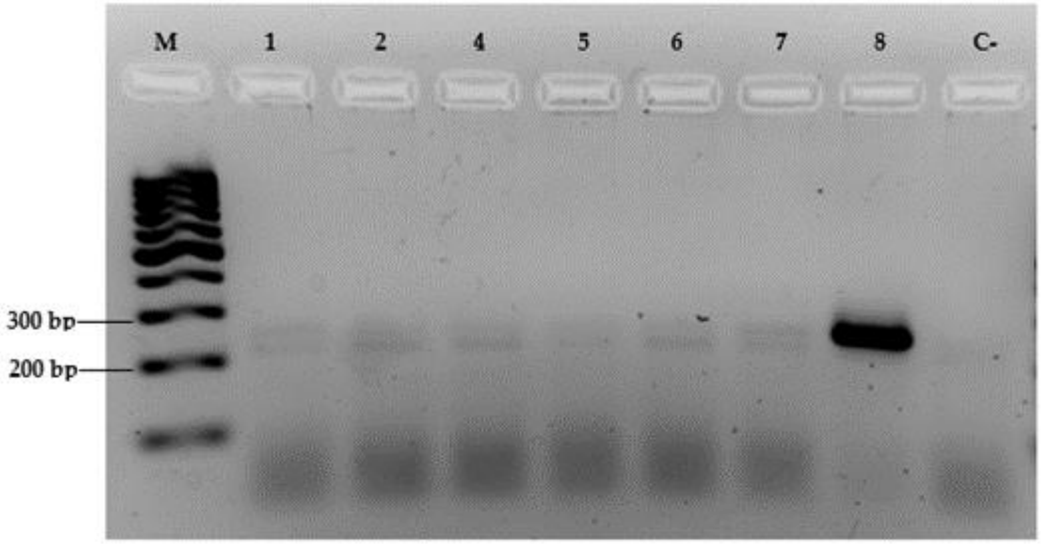

Figure S2. Representative agarose gel electrophoresis (4%) of the PCR from apiary samples. Note that lane 8 shows a single band corresponding to the expected 252 bp of V. ceranae, lane C (-) is the negative control of PCR reaction (no DNA), whereas lanes 1-7 display two PCR products, one of the exact size as lane 8 and a smaller amplicon. M denotes 100 bp ladder.
